# Green Synthesis, Characterization and Application of Proanthocyanidins-Functionalized Gold Nanoparticles

**DOI:** 10.3390/nano8010053

**Published:** 2018-01-21

**Authors:** Linhai Biao, Shengnan Tan, Qinghuan Meng, Jing Gao, Xuewei Zhang, Zhiguo Liu, Yujie Fu

**Affiliations:** 1 Key Laboratory of Forest Plant Ecology, Ministry of Education, Northeast Forestry University, Harbin 150040, China; klp15blh@nefu.edu.cn (L.B.); mengqinghuan@nefu.edu.cn (Q.M.); gjing_123@hotmail.com (J.G.); xueerzh@hotmail.com (X.Z.); 2 State Engineering Laboratory of Bio-Resource Eco-Utilization, Harbin 150040, China; 3 Analysis and Test Center, Northeast Forestry University, Harbin 150040, China; tsnan_99@hotmail.com

**Keywords:** proanthocyanidins, gold nanoparticles, heavy metal ions, dye

## Abstract

Green synthesis of gold nanoparticles using plant extracts is one of the more promising approaches for obtaining environmentally friendly nanomaterials for biological applications and environmental remediation. In this study, proanthocyanidins-functionalized gold nanoparticles were synthesized via a hydrothermal method. The obtained gold nanoparticles were characterized by ultraviolet and visible spectrophotometry (UV-Vis), Fourier transform infrared spectroscopy (FTIR), transmission electron microscopy (TEM) and X-ray diffraction (XRD) measurements. UV-Vis and FTIR results indicated that the obtained products were mainly spherical in shape, and that the phenolic hydroxyl of proanthocyanidins had strong interactions with the gold surface. TEM and XRD determination revealed that the synthesized gold nanoparticles had a highly crystalline structure and good monodispersity. The application of proanthocyanidins-functionalized gold nanoparticles for the removal of dyes and heavy metal ions Ni^2+^, Cu^2+^, Cd^2+^ and Pb^2+^ in an aqueous solution was investigated. The primary results indicate that proanthocyanidins-functionalized gold nanoparticles had high removal rates for the heavy metal ions and dye, which implies that they have potential applications as a new kind of adsorbent for the removal of contaminants in aqueous solution.

## 1. Introduction

Gold nanoparticles are one of the most extensively studied noble metal nanomaterials due to its potential applications in catalysis, optical and electronic devices, biodiagnostics and medicine [1,2,3]. Various wet chemical methods have been reported for the synthesis of gold nanoparticles, such as citrate reduction and the Brust-Schiffrin method [1,4]. It has been discovered that the capping agents or the shells of gold nanoparticles are very important for their physicochemical properties, toxicity and applications [2,5,6]. In addition, the development of novel synthetic strategies for the preparation of monodisperse gold nanoparticles and the assembly of nanoparticles into one- and two-dimensional structures are critical for their applications [7,8]. Recently, there has been a renewed interest in green synthesis of nanoparticles [9,10,11,12,13,14]. For example, gold nanomaterials were synthesized by using plant extracts as both the reducing and capping agents [15]. Plant extracts that have great potential in heavy metal accumulation and detoxification are the best candidates for nanoparticle green synthesis and environmental remediation applications.

Pomegranates are cultivated and consumed in large quantities in China. Pomegranate peels (*Punica granatum*) are usually discarded as waste. However, it has been found that pomegranate peel contains a significant portion of polyphenols and proanthocyanidins, and has the highest antioxidant activity among the peel, pulp and seed fractions [16,17]. The extract yield of polyphenols and proanthocyanidins from pomegranate peels by water extraction was 15–20% and 1.2–9.0%, respectively [16,18]. Polyphenols and proanthocyanidins are well known for their antioxidant activity [19,20,21]. Therefore, the water extract of pomegranate peels could be a potential reducing compound used for the green synthesis of gold nanoparticles.

Green synthesis of metal nanoparticles using plant extracts can minimize their toxicity, whereas most chemical methods for the synthesis by using hazardous compounds such as hydrazine, sodium borohydride, and dimethyl formamide (DMF) as reducing agents results in a more complicated and time-consuming process for removing the toxic compounds for biomedical applications. Furthermore, synthesis of metal nanoparticles using plant extracts is very cost effective, and therefore can be used as an economical and valuable alternative to the large-scale production of metal nanoparticles. In addition, the full utilization of plant waste is a sustainable path for development.

In this study, we explored using the water extract of pomegranate peels as both a reducing and a capping agent to prepare gold nanoparticles via a hydrothermal method. Since proanthocyanidins are the main components of the water extract of pomegranate peels, which are the mixtures of the several different compounds (nearly one hundred phenolic compounds) [17], we further focused on the use of proanthocyanidins for the synthesis of gold nanoparticles.

Proanthocyanidins, naturally occurring antioxidants widely available in vegetables, fruits, nuts, seeds, flowers, and bark, have been reported to possess a broad spectrum of biological, pharmacological, and therapeutic activities against free radicals and oxidative stress [19,22,23,24]. To the best of our knowledge, the green synthesis of proanthocyanidin-functionalized gold nanoparticles has not been thoroughly explored. Furthermore, we investigated the application of proanthocyanidin-functionalized gold nanoparticles for the removal of dyes and heavy metal ions Ni^2+^, Cu^2+^, Cd^2+^ and Pb^2+^ in an aqueous solution. 

## 2. Materials and Methods

### 2.1. Materials

Proanthocyanidins were purchased from the Aladdin Reagent Co., Ltd. (Shanghai, China). Tetrachloroauric (III) acid hydrate was obtained from Sinopharm Chemical Reagent Co., Ltd. (Shanghai, China). All other reagents were of analytical purity grade. Ultra-pure water was used throughout this study, and its resistivity was >18 MΩ·cm.

### 2.2. Synthesis of Pomegranate Peel Extract Protected Gold Nanoparticles

The dried pomegranate peel was ground into a fine powder and collected through an 80-mesh sieve. Pomegranate peel powder was degreased with petroleum ether (solid-liquid ratio 1:20) for 1 h, and then filtrated and dried. A microwave-assisted extraction method was used to extract the pomegranate peel powder. In a conical flask, 2 g of dried peel powder was added to 50 mL of pure water. After extraction by microwave at 350 w for 70 s, the mixed solution was further extracted at 100 °C for 60 min. The mixed solution was then centrifuged at 10,000 rpm for 10 min. The obtained supernatant was used for the following synthesis. The supernatants were diluted by a factor of 40 with pure water. 0.64 mL of 2% HAuCl_4_·4H_2_O aqueous solution was then added to 50 mL of diluted supernatant. The mixed solution was stirred for 30 min. 40 mL of the final solution was transferred into a Teflon-lined 50 mL autoclave, and then sealed and maintained at 140 °C for 4 h. 

### 2.3. Synthesis of Proanthocyanidin-Functionalized Gold Nanoparticles 

In a typical experiment, 50 mL of the 0.64 mM proanthocyanidin aqueous solution was prepared by dissolving proanthocyanidins in pure water. 0.64 mL of the 2% HAuCl_4_·4H_2_O aqueous solution was then added. The mixed solution was stirred for 30 min. 40 mL of the final solution was transferred into a 50 mL Teflon-lined autoclave, and then sealed and maintained at 140 °C for 4 h. A dark red solution was obtained, which indicated that proanthocyanidin-functionalized gold nanoparticles had been produced. The pH of the final gold colloid was approximately 2.5.

### 2.4. Removal of Heavy Metal Ions and Methylene Blue (MB)

To explore the removal effect on heavy metal ions and methylene blue (MB), gold nanoparticles were separated from the initial solution by centrifugation. They were obtained by centrifugation at relative centrifugal force RCF 22,680 g for 15 min. The obtained gold nanoparticles were dried at 60 °C overnight. In a typical experiment, proanthocyanidin-functionalized gold nanoparticles were added to 35 mL of a 20 mg/L heavy metal ion (Ni^2+^, Cu^2+^, Cd^2+^ and Pb^2+^) solution at pH 8.0 or MB solution, 0.0350 g (final concentration of 1 g/L). The mixed solution was shaken at 140 rpm for a predefined time (generally 6 h). Gold nanoparticles were then centrifuged at RCF 22,680 g for 6 min, and an aliquot of the supernatant was used to determine the remaining concentration of the heavy metal ions by flame atomic absorption spectrometry. Based on the obtained concentrations of the heavy metal ions in the supernatant, the removal rate for heavy metal ions by proanthocyanidin-functionalized gold nanoparticles could be calculated. The removal efficiency for heavy metal ions by gold nanoparticles under different pH conditions was also investigated. Gold nanoparticles were added into the 20 mg/L heavy metal ion solution at pHs varying from 4 to 10 for 6 h, and then the same operations were performed as mentioned before.

### 2.5. Characterization of Gold Nanoparticles and Determination of the Concentration of Heavy Metal Ions 

#### 2.5.1. UV-Vis Absorption Spectra

UV absorption spectra of the gold colloid were measured by UV-5500 spectrophotometer (Metash, Shanghai, China). 4 mL of the dilute colloid solution was added into a quartz cuvette for the measurement.

#### 2.5.2. Fourier Transform Infrared Spectroscopy

FTIR spectra were measured by IR Affinity-1 (Shimadzu, Kyoto, Japan) using the KBr pellet method. The sample was prepared by mixing the precipitate of the gold nanoparticles (derived from the centrifugation of the gold colloid solution) with a small amount of solid KBr. FTIR spectra of the pure proanthocyanidins was also measured for comparison.

#### 2.5.3. Transmission Electron Microscopy

The produced gold nanomaterials were imaged using a JEM-2100 transmission electron microscope (JEOL Ltd., Tokyo, Japan) at 200 kV. The samples for TEM characterization were prepared by placing 5 μL of the as-synthesized gold colloid solution on a carbon coated copper grid and dried at room temperature.

#### 2.5.4. X-ray Diffraction

Powder X-ray diffraction patterns were record using D/MAX 2200VPC, Tokyo, Japan) (40 kV/40 mA). The sample for XRD determination was derived from centrifugation of the gold colloid solution.

#### 2.5.5. Atomic Absorption Spectrometry

The metal content was determined by flame atomic absorption spectroscopy equipped with a hollow cathode lamp and an air-acetylene flame (AnalytikJena AG, novAA 350, Jena, Germany). The wavelengths (nm) used for the determination of the analyses were: copper 324.8, nickel 232.0, lead 283.3 and Cadmium 228.8. Gas flow was 50 dm^3^·h^−1^ and the aspiration rate was 5 cm^3^·min^−1^.

## 3. Results and Discussion

### 3.1. UV-Vis and FTIR Absorption Spectra

**Figure 1:**
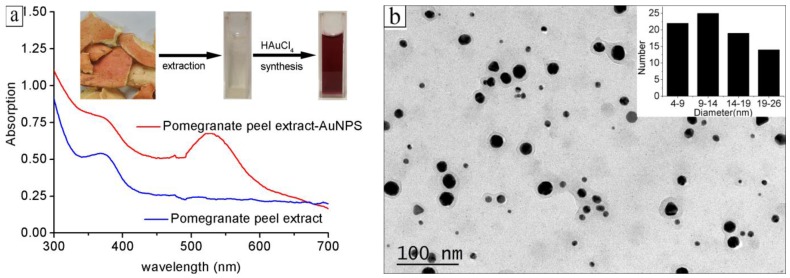
(**a**) UV-Vis absorption spectra of the water extract of pomegranate peels and the synthesized gold colloid in a typical experiment; (**b**) The representative TEM image of the obtained gold nanoparticles.

Figure 1a shows the UV-Vis absorption spectra of the water extract of the pomegranate peels and the synthesized gold colloid in a typical experiment. The water extract of the pomegranate peels had no obvious UV absorption from 400 nm to 700 nm, as indicated in Figure 1a. There is an absorption band peaking at 530 nm after the hydrothermal process, which can be ascribed to the plasmon resonance band (PRB) of the gold nanoparticles [25]. The representative TEM image of the obtained gold nanoparticles is shown in Figure 1b. The size distribution of the gold nanoparticles is indicated in the inset. The average diameter of gold nanoparticles was 13.0 ± 5.4 nm. As shown in Figure 1b, the size distribution of the gold nanoparticles is relatively broad, wherein there are both some large and some small nanoparticles. In order to obtain monodispersed gold nanoparticles, pure proanthocyanidins were investigated in the following synthesis, since proanthocyanidins are the main components of the water extract of pomegranate peels.

**Figure 2:**
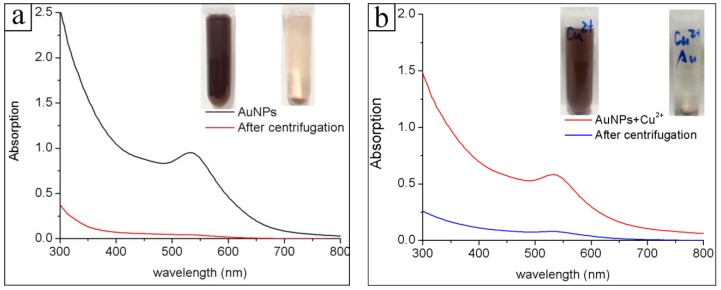
(**a**) UV-Vis absorption spectra of the proanthocyanidin-functionalized gold nanoparticles solution before and after centrifugation; (**b**) UV-Vis absorption spectra of the proanthocyanidin-functionalized gold nanoparticles redispersed in the 20 mg/L CuCl_2_ solution before and after centrifugation.

Figure 2a shows the UV-Vis absorption spectrum of the obtained proanthocyanidins-functionalized gold colloid in a typical experiment. The absorption band peaking at 532 nm is the plasmon resonance band (PRB) of the gold nanoparticles, which is in accordance with the previous studies [25]. The single PRB also indicates that the obtained gold nanomaterials are mainly spherical in shape, in accordance with the Mie theory, and did not form agglomerates [26,27]. In most of the reported cases of green syntheses via plant extracts, the PRBs of the gold nanoparticles were usually broad and weak [21,28]. Here, the strong PRBs were due to the high productivity of gold nanoparticles in the synthesis. The stability of the gold colloid can be monitored by UV-Vis absorption spectra. The obtained gold colloid was very stable during storage, and could be maintained at room temperature for more than one month without obvious changes. In addition, the proanthocyanidin-functionalized gold nanoparticles showed good stability in the different NaCl concentrations (0.1–0.5 M) and pH solutions (3.0–9.0). The stability of the obtained gold nanoparticles was further verified in the following centrifugation-redispersion experiments. The proanthocyanidin-functionalized gold nanoparticles were collected by centrifugation, as indicated in the inset in Figure 2a. They were able to be redispersed and recovered in pure water or a metal salts solution into a stable gold colloid. Figure 2b shows the UV-Vis absorption spectrum of proanthocyanidins-functionalized gold nanoparticles redispersed in the CuCl_2_ solution. The UV-Vis absorption spectrum of the redispersed solution is nearly same as that of the initial solution in the synthesis. Gold nanoparticles can be further collected by centrifugation to test their adsorption ability for heavy metal ions as shown in inset in Figure 2b. It is known that the commonly used gold nanoparticles reduced by citrate or NaBH_4_ method tend to aggregate in the repeat centrifugation-redispersion experiments. The extra stability of proanthocyanidins-functionalized gold nanoparticles in this study implied that the surfaces of the gold nanoparticles were well protected by the associated proanthocyanidins.

**Figure 3:**
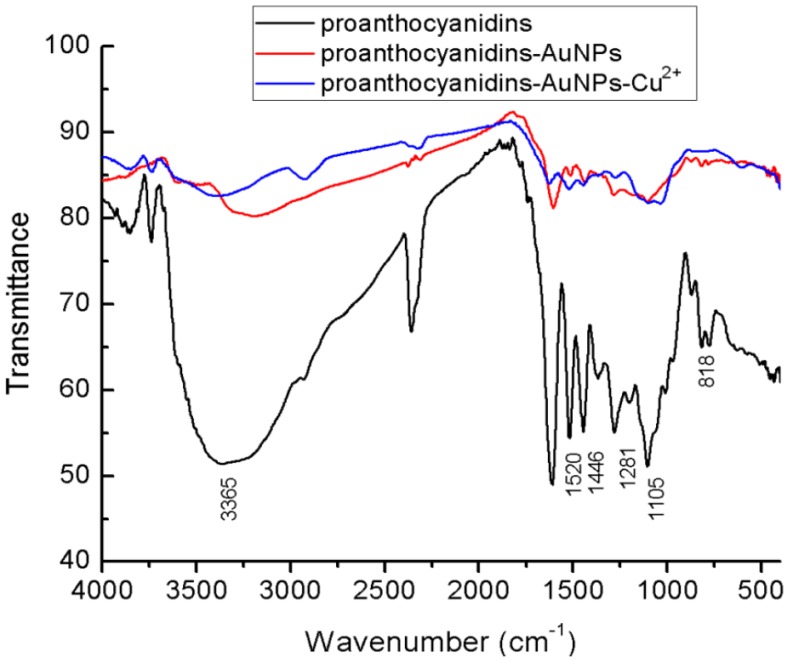
FTIR absorption spectra of pure proanthocyanidins and the proanthocyanidin-functionalized gold nanoparticles.

Figure 3 shows the FTIR spectrum of the pure proanthocyanidins and proanthocyanidins-functionalized gold nanoparticles. In the spectrum of pure proanthocyanidins, the broad absorption band peaking at 3365 cm^−1^ was ascribed to the formation of the hydrogen bond between phenolic hydroxyl of proanthocyanidins. The absorption bands at 1610 and 1105 cm^−1^ were attributed to those of the characteristic functional groups of poly flavonoids moiety in proanthocyanidins, whereas those at 1520 and 777 cm^−1^ were attributed to the skeletal stretching modes of the aromatic ring and CH out-of-plane deformation of the aromatic rings with two adjacent free hydrogen atoms, respectively, indicating the prominent presence of procyanidin (PC) structure [29,30]. In the FTIR spectrum of proanthocyanidins-functionalized gold nanoparticles, the characteristic absorption bands were quite similar to those of standard proanthocyanidins, except that the absorption band corresponding to the hydrogen bond moved to 3200 cm^−1^. These results confirmed that proanthocyanidins were associated with gold nanoparticles. The shift effect at the hydrogen bond region implied that phenolic hydroxyl of the proanthocyanidins had strong interactions with the surface of the gold nanoparticles.

The FTIR spectrum of the proanthocyanidin-functionalized gold nanoparticles after adsorption of Cu^2+^ is also shown in Figure 3. The characteristic absorption bands were quite similar to those of the standard proanthocyanidins and proanthocyanidin-functionalized gold nanoparticles without Cu^2+^, except that the absorption band corresponding to the hydrogen bond moved to 3420 cm^−1^. This result can be ascribed to the coordination effect of Cu^2+^ with phenolic hydroxyl of proanthocyanidin, which influenced their hydrogen bond interactions.

### 3.2. TEM and XRD Characterization 

**Figure 4:**
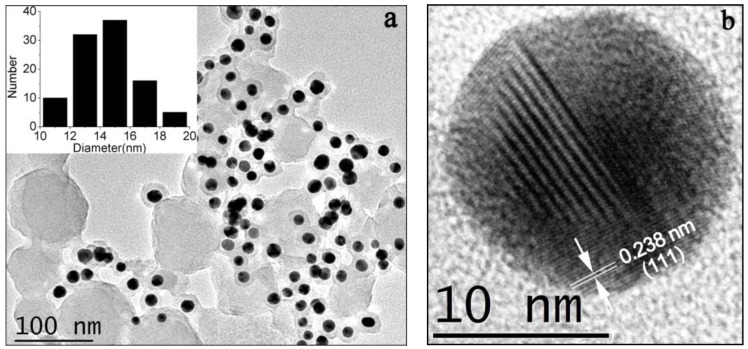
(**a**) The representative TEM images of proanthocyanidin-functionalized gold nanoparticles; (**b**) HRTEM images of one typical gold nanoparticle.

Figure 4a shows the representative TEM image of the gold nanoparticles. Most of the gold nanoparticles were of spherical shape with a narrow size distribution. It has been noticed that some gold nanoparticles shared their proanthocyanidin shell, and formed a structure like a plum pudding model, as indicated in Figure 4a. The size distribution of the gold nanoparticles is indicated in the inset. The average diameter of the gold nanoparticles is 14.5 ± 2.0 nm (*n* = 100). Gold nanoparticles with different size can be obtained by using a seed growth method. For example, the as-prepared gold nanoparticles in the typical experiment at 140 °C were added as seeds to the synthesis solution, then heated to 140 °C for 4 h. The average size of the obtained gold nanoparticle is 28 nm, which is almost two times the mean size of the seeds. A high resolution transmission electron microscopy HRTEM image of one typical spherical gold nanoparticle is shown in Figure 4b. The lattice fringe spacing indicated in the image is 0.238 nm, which corresponds to the (111) facet of crystal plane of the gold cubic phase. 

**Figure 5:**
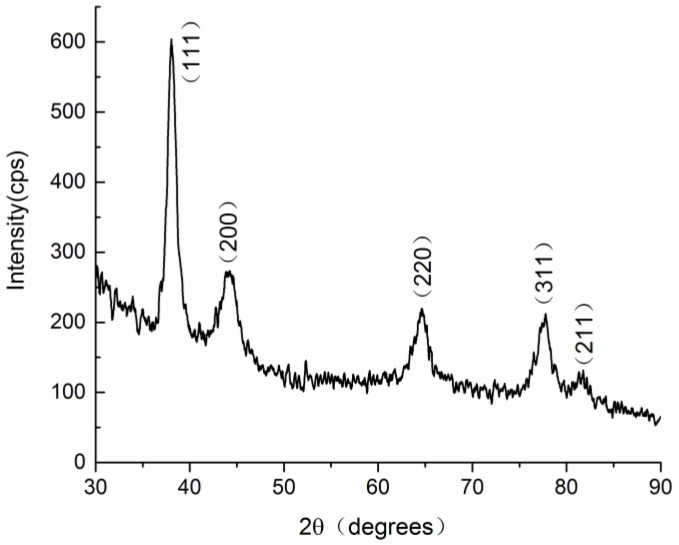
XRD patterns of the proanthocyanidin-functionalized gold nanoparticles.

The crystalline nature of the gold nanoparticles was further verified by the XRD measurement, as shown in Figure 5. The peaks were assigned to diffractions from the (111), (200), (220) and (311) planes of face centered cubic (fcc) gold (JCPDS 04-0784), respectively [27]. This result indicates that the nanoparticles are pure, well-crystallized gold crystals. The width of the (111) peak was employed to calculate the average crystallite size using the Scherrer equation. The calculated average size is 10.1 nm, which is a little smaller than the particle size obtained from TEM. A similar difference between TEM and XRD determination of nanomaterials has also been reported in a recent study [31].

### 3.3. Formation Mechanism of Proanthocyanidins-Functionalized Gold Nanoparticles

In this study, oligomeric proanthocyanidin complexes (OPCs, dimeric, trimeric and tetrameric proanthocyanidin) were used in the synthesis. OPCs are primarily known for their antioxidant activity against free radicals and oxidative stress [32]. Thus, proanthocyanidin can be considered as a class of reducing agents. The reaction for the formation of gold nanoparticles is believed to be a typical redox process, wherein the gold ions are reduced to gold atoms by proanthocyanidins. Proanthocyanidins are comprised of the monomeric unit flavan-3-ol (+) catechin and (−) epicatechin. The multiple hydroxyl groups (–OH) from the basic monomer catechin and epicatechin are the potential reactive sites for reducing Au(III) in the synthesis [21]. The initial step of oxidation may occur on the B-ring of the catechin due to partial deprotonation, which leads to the transformation of *o*-phenols to *o*-quinones, a fundamental step in browning. *O*-quinones are highly reactive species that form dimers and subsequently polymers due to prolonged autoxidation with other polyphenolic molecules [33]. At the same time, the Gold (III) was reduced to atomic Au. Some additional hydroxyl groups from the catechin and epicatechin interacted with the gold surface, as revealed by FTIR determination, which was finally made up of the functionalized shell of the gold nanoparticles.

### 3.4. Removal of Dye and Heavy Metal Ions in Aqueous Solution

**Figure 6:**
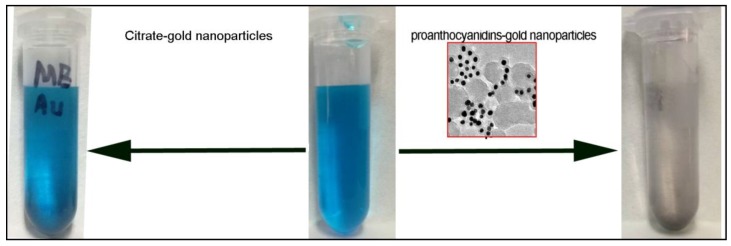
Photograph of the methylene blue (MB) solution (20 mg/L) before addition of gold nanoparticles and after addition of gold nanoparticles for 30 min.

The discharge of various dyes into the hydrosphere is a significant source of water pollution, due to their recalcitrant nature. Methylene blue (MB), a heterocyclic aromatic chemical compound, is extensively used in the textile industry to colorize products, which can be considered to be one kind of typical dye discharged into the environment [34,35]. MB is the most commonly used substance for dying cotton, wood and silk. Various materials, such as natural materials, industrial solid wastes, agricultural by-products, and biosorbents, have been developed in the removal of MB from wastewater [36,37,38]. In this study, the removal of MB in water solution by proanthocyanidins-functionalized gold nanoparticles was investigated. Figure 6 shows a photograph of the 20 mg/L MB solution, and the removal effects of the addition of proanthocyanidin-functionalized gold nanoparticles (initial concentration, 1 g/L). Citrate-gold nanoparticles were also tested as the control sample, as indicated in Figure 6. The solution color did not change with the addition of citrate-gold nanoparticles. In addition, it has been reported that the mixing of the citrate-gold nanoparticles with methylene blue (MB) enhanced the extinction coefficient and absorbance of the dye [39,40]. However, the blue solution was decolored by proanthocyanidin-functionalized gold nanoparticles within 30 min, as shown in Figure 6. The removal rate was more than 98% as measured from UV absorption at 664 nm. The adsorption mechanism is believed to be related with the interactions between proanthocyanidins on the gold nanoparticle surface and molecular moiety of MB. Methylene blue molecules are likely attracted to the nanoparticle surface by dipole-dipole interactions between the nitrogen in the methylene blue and the phenolic groups of proanthocyanidins and their chemical association.

In addition to the dyes, we further explored proanthocyanidin-functionalized gold nanoparticles for the removal of heavy metal ions in aqueous solutions. Toxic heavy metal ions have been excessively released into the aquatic environment due to various industrial activities, and have created a major global concern [41]. The major toxic metal ions in water that are hazardous to humans, as well as other forms of life, are cadmium, copper, nickel, lead, mercury and chromium. Conventional methods for the removal of metal ions from aqueous solutions include chemical precipitation, ion exchangers, chemical oxidation/reduction, reverse osmosis, electrodialysis, ultrafiltration, etc. [41,42]. However, these conventional techniques have their own inherent limitations. It is, therefore, necessary to develop new methods or nanomaterials for the low-cost, high-efficiency minimization of chemical or biological sludge and the regeneration of biosorbents.

Gold nanoparticles have been used for a variety of applications, such as in catalysis, optical and electronic devices, biodiagnostics and bioimaging [2,43]. However, there are few studies on the application of gold nanoparticles for the removal of heavy metal ions. The gold nanoparticles in this study were functionalized by oligomeric proanthocyanidin complexes, which are also very potent metal-chelating agents derived from the multiple hydroxyl groups (–OH) of catechin and epicatechin in proanthocyanidin [22,33]. Therefore, we further investigated the application of proanthocyanidin-functionalized gold nanoparticles on the treatment of the typical heavy metal ions Cu^2+^, Cd^2+^, Ni^2+^ and Pb^2+^.

**Figure 7:**
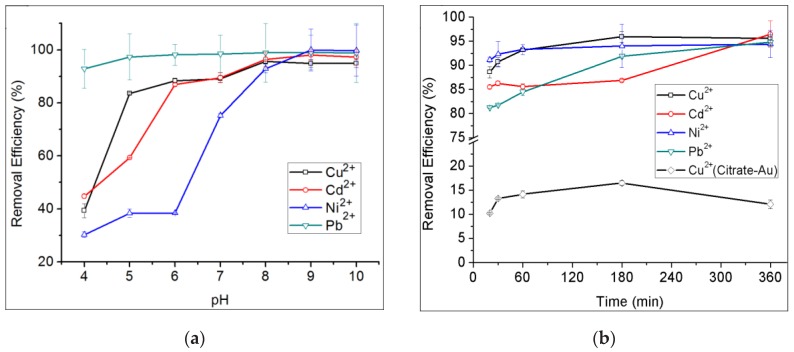
(**a**) The removal efficiency of proanthocyanidin-gold nanoparticles for 20 mg/L of Cu^2+^, Cd^2+^, Ni^2+^ and Pb^2+^ at pH 4.0–10.0 after incubation for 6 h; (**b**) the removal efficiency of proanthocyanidin-gold nanoparticles for 20 mg/L of Cu^2+^, Cd^2+^, Ni^2+^ and Pb^2+^ at adsorption time from 20 min to 360 min under pH 8.0. The removal efficiency of citrate-gold nanoparticles for the removal of Cu^2+^ was also tested for comparison.

The removal efficiency of proanthocyanidins-functionalized gold nanoparticles for heavy metal ions under different conditions was evaluated. It was found that the pH and adsorption time are the two critical factors influencing the removal efficiency for heavy metal ions. Figure 7a shows the removal efficiency of gold nanoparticles for Cu^2+^, Cd^2+^, Ni^2+^ and Pb^2+^ at different pH conditions after incubation in the corresponding metal salts solution for 6 h. The results indicated that the removal efficiency increased as pH rose from 4.0 to 8.0, and was nearly constant at high levels of pH 8.0 from 10.0. With a pH of 8.0, the removal rates for Cu^2+^, Cd^2+^, Ni^2+^ and Pb^2+^ were 95.6%, 96.5%, 92.9% and 98.9%, respectively. Since a pH of 8.0 is a more moderate condition, it was chosen to be used in the next experiments. Figure 7b shows the removal efficiency of gold nanoparticles for Cu^2+^, Cd^2+^, Ni^2+^ and Pb^2+^ under different adsorption times at pH 8.0. The results revealed that the adsorption process was very quick at the initial stage, gradually slowing down after 30 min incubation, and was then constant from 3 h to 6 h. For instance, the removal rate for Cu^2+^ was 81.4% after 1 min adsorption, and more than 95% after 3 h incubation. The adsorption characteristics of gold nanoparticles for Cd^2+^, Ni^2+^ and Pb^2+^ were also very similar to that for Cu^2+^. In summary, the optimized adsorption condition for Cu^2+^, Cd^2+^, Ni^2+^ and Pb^2+^ by gold nanoparticles is a pH of 8.0, with an adsorption time of 3–6 h. Control experiments using citrate gold nanoparticles for the removal of Cu^2+^ were also performed. The highest removal rate by citrate gold nanoparticles for Cu^2+^ was only 16.5%, as indicated in Figure 7b. 

One advantage of proanthocyanidin-functionalized gold nanoparticles in removing heavy metal ions is that gold nanoparticles are easy to collect from the aqueous solution by centrifugation, as indicated in Figure 2b, and then for recovery for further utilization. Nearly all of the gold nanoparticles were able to be collected from the metal salts solution, as judged by the solution color and UV absorption spectra. The superior stability of proanthocyanidin-functionalized gold nanoparticles in repeat centrifugation-redispersion experiments implied that the surface of gold nanoparticles was well protected by the associated proanthocyanidins. In addition, some gold nanoparticles are able to share their proanthocyanidins shell, as indicated in Figure 4a, and form a plum pudding model structure, which may contribute to their characteristic stability and easy manipulation.

**Figure 8:**
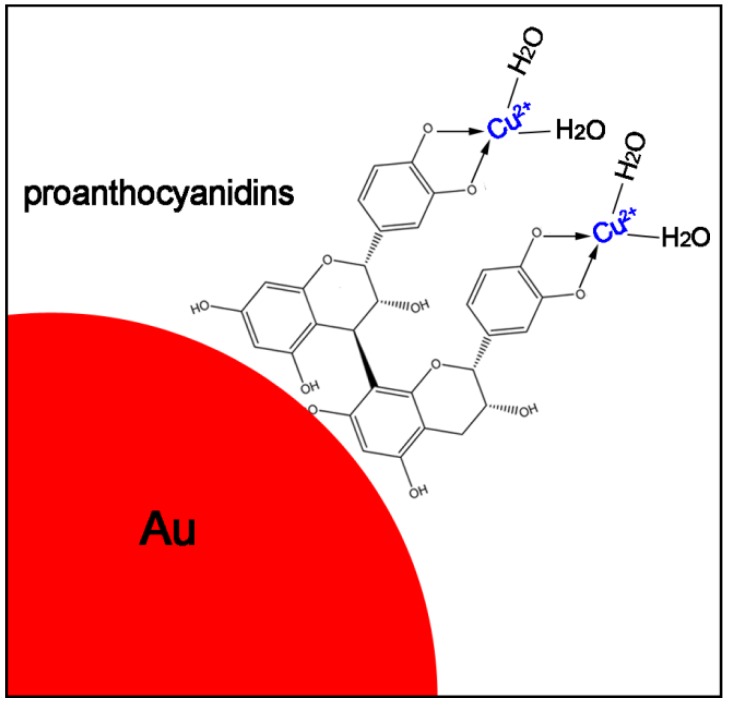
Schematic illustration of the interaction process between proanthocyanidin-functionalized gold nanoparticles and heavy metal ions (Example for Cu^2+^).

The adsorption of heavy metal ions by proanthocyanidin-functionalized gold nanoparticles can be ascribed to the proanthocyanidin shell, since the PRB band of gold nanoparticles did not show obvious changes when they were redispersed in the heavy metal ion solution. The interaction between metal ions and the remaining hydroxyl groups (–OH) of proanthocyanidin on the gold surface plays an important role in sequestering the metal ions. The adsorption process can be schematically illustrated in Figure 8. Oligomeric proanthocyanidin complexes were well covered on the gold nanoparticle surface, the remaining hydroxyl groups (–OH) of catechin and epicatechin in proanthocyanidin interacted with the metal ions and acted as a bidentate ligand to coordinate with the heavy metal ions. For example, recent spectroscopic determinations have confirmed that the Cu(II)-catechin complex formation in which Cu(II) ions can react with catechin in the presence of oxygen in a metal chelation and the oxidation process [22].

Recently, Zero valent iron nanoparticles (nZVI) have been explored for the removal of heavy metal ions in groundwater and wastewater [44]. The mechanisms that facilitate the removal are complex. It has been speculated that the core-shell structure in the iron nanoparticles involve the removal processes wherein the metallic iron core acts as an electron donor source, promoting reduction of the compounds. The shell layer, generally composed of Fe^2+^, Fe^3+^ and O, enables sorption, surface complexation, and electron transport from and to the core. A variety of heavy metal ions can be removed by nZVI with high rates and capacities [44,45]. However, there are some disadvantages to using nZVI, such as the fact that some of the metal ions can be released from the nZVI over long periods of time [44]. In comparison to the nZVI system, the removal efficiency of gold nanoparticles for heavy metal ions is higher in this study [45]. Furthermore, there were no re-dissolving phenomena for at least 12 h in the proanthocyanidins-functionalized gold system. Thus, proanthocyanidins-functionalized gold nanoparticles are expected to be developed as a new kind of adsorbent to remove the heavy metal ions in groundwater and wastewater. 

## 4. Conclusions

A facile, one-step hydrothermal method has been developed to prepare proanthocyanidin-functionalized gold nanoparticles. The gold nanoparticles have good mono-dispersity and a highly crystalline structure. Furthermore, the proanthocyanidin-functionalized gold colloid was very stable, and could be repeatedly collected and redispersed in the aqueous solution without obvious changes. Most importantly, proanthocyanidin-functionalized gold nanoparticles can be utilized for the removal of dyes and heavy metal ions in the aqueous solution. The primary results demonstrated that proanthocyanidin-functionalized gold nanoparticles have high removal rates for dye and heavy metal ions, which implies that they have potential applications as a new kind of adsorbent for the removal of contaminants in an aqueous solution.
